# Effectiveness of emotional freedom techniques (EFT) vs sleep hygiene education group therapy (SHE) in management of sleep disorders among elderly

**DOI:** 10.1038/s41598-022-10456-w

**Published:** 2022-04-20

**Authors:** Nagwa Souilm, Nancy Mahmoud Elsakhy, Yasir A. Alotaibi, Safaa Abdelazem Osman Ali

**Affiliations:** 1 Faculty of Nursing, Beni-Suef University, Beni-Suef, Egypt; 2 Faculty of Nursing, Matrouh University, Matrouh, Egypt; 3 Nursing Department, College of Applied Medical Sciences, University of Bisha, Bisha, 61922 Saudi Arabia; 4 Faculty of Nursing, Suez Canal University, Ismailia, Egypt

**Keywords:** Neuroscience, Psychology, Diseases, Health care, Medical research, Neurology, Risk factors, Signs and symptoms

## Abstract

Sleep disorders are common among elderly persons, with deleterious effects on their physical and mental health. Many approaches are used to manage such disorders. To compare the Emotional Freedom Techniques–Insomnia (EFT-I) and Sleep Hygiene Education (SHE) group therapy as two treatments for insomnia in a geriatric population when delivered, and their effects on sleep quality, depression, and life satisfaction. This open-label randomized controlled trial study was conducted at El-Abbasia Mental Hospital and Osana family wellness elderly nursing home at Maadi, Cairo. It included 60 elderly patients suffering insomnia sleep problems randomized into two equal groups: one group received a Sleep Hygiene Education (SHE) intervention, the other had a form of Emotional Freedom Techniques (EFT) adapted for use with insomnia (EFT-I). A self-administered questionnaire with tools for sleep quality (*Pittsburgh Sleep Quality Index [PSQI])*, depression, and life satisfaction was used to collect data. The fieldwork was from January to March 2021. The two groups had equal median age (70 years), and almost similar gender and place of residence distribution. After the intervention, 73.3% of the EFT group had good sleep quality, compared to 100.0% in the SHE group (*P* = 0.005); the median score of depression (3.00) was higher in the EFT group compared with 0.00 in the SHE group (*P* < 0.001); as for life satisfaction, the difference was not statistically significant. The multivariate analyses identified the study intervention as the main statistically significant negative predictor of PSQI and depression scores, and a positive predictor of life satisfaction. Being in the SHE group was a negative predictor of PSQI and depression scores. Both SHE and EFT approaches are beneficial for elderly patients’ sleep quality, with SHE being more effective in ameliorating sleep. Further replication of this study is needed on a large probability sample from different geographical areas to help for the generalization of the results.

## Introduction

Sleep is a physiological function that is essential for life and survival^1^*.* Sleep disorders are common in the general population, and especially in the geriatric age group, and the problem has escalated with the COVID-19 pandemic^2–4^. There is a tendency for sleep patterns to change with aging. Elder persons mostly have trouble with sleep, particularly with falling asleep. They tend to have frequent night wakeups, 3–4 times per night, and in addition, they have early morning waking. Their total duration of sleep may also decrease slightly. Elderly people have a less dreamless or deep sleep. They feel they have lighter sleep in comparison to earlier age time, and this could be attributed to the often feeling of rapid switch between sleep and wakeup, and their awareness of being awake is high^5^. Such poor sleep has been associated with low physical performance among elders^6^.

The sleep disorders associated with older age could have several underlying causes. Among others are the more frequent need for micturition at night or nocturia, and pain or discomfort due to commonly existing chronic diseases, especially diabetes^6^. Moreover, staying in bed for a long time could lead to more difficulty in falling asleep. These factors deprive elderly people of the benefits of good sleep with “delta waves,” which have been shown to have a restorative effect on body functions. Additionally, being deep, such sleep can decrease the chances of waking up from environmental sleep-disturbing factors like noise, light, heat, or cold, as well as pain and discomfort. Conversely, disturbed and particularly short-duration sleep has been associated with mental illnesses and Alzheimer’s disease^7,8^.

Non-pharmacological management of sleep disorders has always been recommended particularly in the geriatric population^9^. “Sleep hygiene,” term introduced by Hauri^10^ as an approach to help insomnia patients improve their sleep. It refers to a combination of sleep habits shown to help such patients have the best possible sleep quality. These involve certain lifestyle changes such as having dinner at least three hours before bed, as well as avoiding heavy meals and excess sweets before sleep^11^. Caffeinated beverages should be restricted for at least six hours before bedtime. Light physical exercises help faster initiation of sleep^12^. The use of electronic screens such as TV or mobile phones disturbs the normal secretion of melatonin, leading to sleep disturbance, and thus should be avoided one hour before bedtime. A quiet environment with a comfortable temperature is also important. Additionally, minimizing stress, with meditation and relaxation exercises are efficient promoters of sleep quality^13^.

Another approach to manage sleep disorders is the Emotional Freedom Technique (EFT). A version of this is dedicated to insomnia (EFT-I). The technique uses tapping to help a person to relax and fall asleep by releasing energy and relieving stress. It helps get rid of the individual’s concerns about the inability to fall asleep, and of various stressors that may lead to sleep disorders^14^.

### Significance of the study

Sleep disorders are common among elderly persons, which may have deleterious effects on their physical and mental health. There are many approaches used to manage such disorders, with varying outcomes. Two main approaches often used are Sleep Hygiene Education (SHE) and Emotional Freedom Techniques (EFT). The merits of these two approaches were seldom compared, especially when administered as group therapy.

### Aim of the study

To compare the Emotional Freedom Techniques–Insomnia (EFT-I) and Sleep Hygiene Education (SHE) group therapy as two treatments for insomnia in a geriatric population when delivered as group therapy, and their effects on sleep quality, depression, and life satisfaction.

### Research hypotheses

(1) Elderly patients receiving SHE intervention will have better sleep quality in comparison with those receiving EFT-I intervention; (2) Elderly patients receiving SHE intervention will have less depression in comparison with those receiving EFT-I intervention; (3) Elderly patients receiving SHE intervention will have better life satisfaction in comparison with those receiving EFT-I intervention.

## Participants and methods

### Research design

An open-label randomized controlled trial design was utilized in this study. It was open-label because the type of intervention could not be concealed from participants or researchers.

### Setting

The study was conducted at El-Abbasia Mental Hospital and Osana family wellness elderly nursing home at Maadi, Cairo. El-Abbasia Hospital for Mental Health is a governmental hospital with a 500-bed capacity. It is the largest hospital for psychiatric patients in Egypt. The hospital provides care for all sectors in the Egyptian community, especially in Cairo. Osana home provides holistic and healing activities for people of all ages. These include yoga, Pilates, and other client-based barefoot classes, as well as treatments such as homeopathy, massage, and emotional freedom technique. It also offers pre and postnatal workshops, classes, and support, in addition to baby, child, and family classes.

### Participants

**Figure 1:**
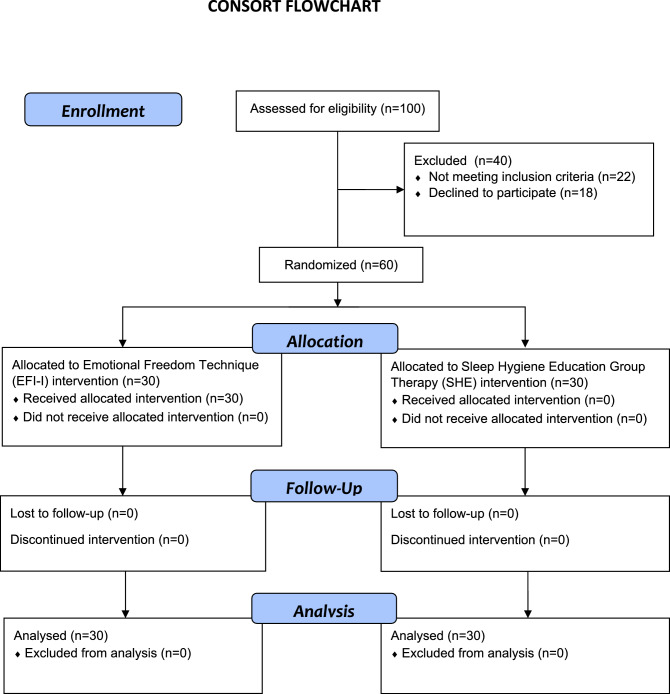
The flowchart of the study.

The study sample consisted of 60 elderly patients suffering from insomnia sleep problems. Those having major physical or psychiatric ailments or being on medication affecting their sleep were excluded. These involved those with a history of epilepsy, seizures, or dementia, current alcohol or substance abuse/dependence (must have > 90 days of sobriety), night shift workers, as well as those unable to complete the study questionnaires and psychological tests. The sample size was calculated to demonstrate a higher percentage of improvement of sleep quality in the sleep hygiene group in comparison with the EFT group, with a relative risk of 1.75 or higher at a 95% level of confidence and 80% power. The required sample size as calculated using the Open-Epi statistical software package was 24 per group. This was increased to 30 per group to compensate for any dropouts or loss to follow-up. The flowchart of the study was presented in Fig. 1.

Patients were randomized into two equal groups of 30 participants each. One group received a Sleep Hygiene Education (SHE) intervention, while the other had a form of Emotional Freedom Techniques (EFT) adapted for use with insomnia (EFT-I).

### Data collection tools

A self-administered questionnaire including a demographic characteristics section and three standardized tools for assessment of sleep quality, depression, and life satisfaction were used to collect the data for the study. The demographic characteristics section was developed by the researchers. Data regarding patient age, gender, marital status, place of residence, educational level, current job status, income, and status of living were collected. The researchers were not blinded during data collection and computational analysis. Blinding was used during the analysis.

The first tool was the *Pittsburgh Sleep Quality Index (PSQI)* is a self-report questionnaire developed by Buysse et al.^15^ to assess sleep quality over a 1-month time interval. The tool filling can be completed in 5–10 min. It is intended to be used as a standardized sleep questionnaire for clinicians and researchers and has been used in many settings for the diagnosis of sleep disorders. Research provides evidence of its validity and reliability^16,17^. The Arabic version of this tool was used in the present study^18^.

The tool consists of 19 items from which seven components covering different aspects of sleep are computed to produce one composite global score. These are sleep latency asking about how long it takes to fall asleep, sleep duration, habitual sleep efficiency measuring the percentage of sleep time of total bedtime, sleep disturbances, use of sleeping medication, daytime dysfunction, in addition to overall subjective sleep quality. Each item is weighted on a 0–3 interval scale, with a higher score indicating worse quality. The global PSQI score is then calculated by totaling the seven component scores, providing an overall score ranging from 0 to 21, where lower scores denote a healthier sleep quality. For categorical analysis, the total score is dichotomized into good sleep quality (total score <  = 5), and poor sleep quality (total score > 5)^15^.

The second tool was the *Geriatric Depression Scale 15-item version (GDS-15).* The original form of the scale was developed by Yesavage et al.^19^, as a screening test for the detection of depression symptoms among elder persons. It also helps in the assessment of the severity of these symptoms and treatment follow-up. It had 30 items and needed a long time for filling. Thus, a newer shorter version *(GDS-15)* was proposed by Sheikh and Yesavage^20^, and its validity was put in evidence^21^.

The tool has 15 items such as: “*Are you basically satisfied with your life?”, “Do you often get bored?”,” Do you feel happy most of the time?”, “Do you think that most people are better off than you are?*”. The response to each item is either Yes or No. These are scored 1 and zero respectively. The scoring was reversed for positive items so that a higher score indicates more severe depression. The scores of the items are summed up giving a total score ranging from 0 to 15. For categorical analysis, the total score is dichotomized into no depression (total score <  = 5), and depression (total score > 5). The validated Arabic version of this tool was used in the present study^22^.

The third tool was the *Satisfaction with Life (SWL)* scale. This tool was developed by Diener et al.^23^, to assess a person’s overall subjective feeling of satisfaction with his/her life. The tool consists of five items such as “*In most ways, my life is close to my ideal*”, “*If I could live my life over, I would change almost nothing*.” The responses are on a 7-point Likert type scale ranging from “strongly disagree’ to “strongly agree.” These are scored from one to seven. The scores of the items are summed up giving a total score ranging from 5 to 35. For categorical analysis, the total score is dichotomized into dissatisfied (total score 5–20), and satisfied (total score 21–35). Research demonstrated high tool validity and reliability^24,25^. Arabic version of this tool was used in the present study^26^.

### Validity and reliability of the tools

The three tools used for data collection are standardized, with evidence of high validity and reliability as indicated in their aforementioned description. Additionally, validated Arabic versions of these tools were used. Hence, they did not need any further validation. However, the prepared self-administered questionnaire with the demographic section and the three tools was presented to five experts in psychiatric nursing to review the demographic part and to arbitrate the relevance of the three tools for the study aim. Moreover, the three tools’ reliability was assessed by examining their internal consistency. They demonstrated good levels of reliability with Cronbach’s alpha coefficients 0.95, 0.60, and 0.87 for the PSQI, depression, and life satisfaction tools respectively.

### Administrative and ethical considerations

Official permissions to conduct this study were obtained from the administration of the study settings through letters issued from the Dean of the Faculty of Nursing, Beni-Suef University. The letter explained the aim of the study and its procedures, as well as a copy of the data collection form.

The current study was approved by the Ethical Committee at the Faculty of Medicine, Alexandria University, Egypt; approval number (0305317). The clinical trial was registered in the “Clinical Trials.gov Protocol Registration and Results System (PRS)”; registration number is (0305317/2021) and the full date of first registration is 19/02/2022. All research ethics were followed in conducting the study according to Helsinki Declaration. The aim of the study and its procedures were explained to elderly patients. They were invited to participate after being informed about their rights to refuse or withdraw from the study at any time without giving any reason. They were reassured that any obtained information would be confidential and used for research purposes only. Informed, verbal consent from all the elderly patients was obtained at recruitment. In the Egyptian culture, psychological disorders are still considered a stigma, so the patient and/or his family would prefer to provide oral consent rather than signing a form. As a result of the significant disruption that is being caused by the COVID-19 pandemic, the researchers were very keen to implement all the WHO guidelines related to the protection against COVID 19 while implementing the clinical trial such as; maintaining social distance, use of face masks, and disinfection solutions. The study maneuvers could not lead to any actual or potential harm to participants.

### Pilot study

A pilot study was conducted on six patients representing 10% of the computed sample size to test the clarity of the data collection form and the feasibility of the research process. Needed modifications were carried out based on the pilot study results, and the tool was finalized accordingly. The patients involved in the pilot were excluded from the study to avoid contamination of the study sample.

### Fieldwork

The study was carried out through assessment, planning, implementation, and evaluation phases.

#### Assessment phase

The 60 participants were recruited according to the inclusion and exclusion criteria. They were examined by a neuropsychiatrist to ensure their diagnosis of either insomnia or depression. They were then asked to fill out the self-administered questionnaire including the three scales, which were considered the baseline or pretest data. Each participant took 30–40 min to complete the questionnaire. They were then randomly assigned to either the EFT or SHE groups using a simple random method.

#### Planning phase

During this phase, the researchers prepared the study intervention programs to be implemented in each of the two groups. This was based on a review of pertinent literature^27–29^. It also took consideration of the participants’ needs identified in the assessment phase.

#### Implementation phase

The intervention programs were administered in the form of instructional guidelines through eight sessions for each of the two groups. Each session duration was 60 min.

- Emotional Freedom Techniques–Insomnia (EFT-I) program: In the first session, the researchers gave a presentation of the program’s aim and objectives, its sessions, as well as the procedures to be followed throughout the intervention. The second session focused on knowledge about insomnia in the elderly, covering its meaning, signs and symptoms, types, and causes. The third session discussed the basics of EFT and its concept of combining elements of cognitive restructuring and exposure techniques with acupoint stimulation through tapping exercises. The fourth, fifth, sixth, and seventh sessions were practical sessions where the researchers trained the participants to focus on a specific matter while tapping on endpoints of the body’s energy meridians. These include the top of the head, eyebrows, under and side of eyes, chin, collarbone, and under the arms. While tapping, the person has to recite certain phrases targeting an emotional element of a physical symptom. The eighth session was a recapitulation of the program and ended with evaluation through post-testing.
- Sleep Hygiene Education (SHE) program: The first and second sessions were similar to those in the EFT group. The third session addressed the concept and basics of SHE. The fourth to seventh sessions were geared towards the education of the participants and training them in healthy sleep habits. They were encouraged to follow a set of advice to improve their sleep, such as avoidance of caffeinated beverages, regular exercise, ensuring a quiet sleeping environment, as well as maintaining a regular sleep schedule. The eighth session was a recapitulation of the program and ended with evaluation through post-testing.

The interventions were delivered in each study group in small groups. The 60-min sessions were administered twice per week for 4 weeks. The researchers attended the previously mentioned study settings two days per week from 9 am to 12 pm for the preparation of the sessions and the small groups. The participants in each group were instructed to use self-treatment and repetitive learning using a cassette tape and recorder. They were asked to listen and follow instructions at least once per day.

#### Evaluation phase

In this phase, a post-test was carried out to assess the effectiveness of the implemented interventions. This was done at the end of the implementation phase using the same self-administered form utilized in the pretest. The four phases of data collection took 3 months, from January to March 2021.

### Statistical analysis

Version 20 of SPSS was used for data entry and analysis. For the qualitative variables, descriptive statistics were used in the form of frequencies and percentages, meanwhile, in quantitative variables, means and standard deviations and medians were used. For assessing reliability, we calculated the Cronbach alpha coefficient. When appropriate, Student t-tests or non-parametric Mann–Whitney tests were used to compare quantitative continuous data. Meanwhile, a test of chi-square or Fisher was used for comparing qualitative categorical variables. We used Spearman rank correlation to assess the inter-relationships between quantitative variables and ranked ones. The independent predictors of PSQI scores, depression, life satisfaction were identified by multiple linear regression analyses. *P*-value < 0.05 was set as the level of statistical significance.

## Results

As illustrated in Table 1, the elderly patients in the EFT and Sleep hygiene groups had equal median age (70 years), and almost similar gender and place of residence distribution. All of the EFT and 96.7% of the Sleep Hygiene group had no current job. Those in this latter group had higher percentages of those carrying a university degree (53.3%), having sufficient income (63.3%), and living with family (86.7%). The corresponding figures in the EFT group were respectively 23.3%, 33.3%, and 63.3%. These differences were statistically significant.

**Table 1:** Socio-demographic characteristics of elderly patients in the two study groups.

	Group				X^2^ test	*p*-value
	EFT (*n* = 30)		Sleep hygiene (*n* = 30			
	No	%	No	%		
**Age**						
<70	10	33.3	13	43.3		
70+	20	66.7	17	56.7	0.63	0.43
Range	59.0–79.0		64.0–78.0			
Mean ± SD	70.2 ± 3.8		70.4 ± 3.8		t = 0.01	0.94
Median	70.0		70.0			
**Gender**						
Male	17	56.7	16	53.3		
Female	13	43.3	14	46.7	0.07	0.80
**Marital status**						
Unmarried	21	70.0	14	46.7		
Married	9	30.0	16	53.3	3.36	0.07
**Residence**						
Urban	14	46.7	13	43.3		
Rural	16	53.3	17	56.7	0.07	0.80
**University degree**						
No	23	76.7	14	46.7		
Yes	7	23.3	16	53.3	5.71	0.02*
**Current job**						
None	30	100.0	29	96.7		
Working	0	0.0	1	3.3	Fisher	1.00
**Income**						
Insufficient	20	66.7	11	36.7		
Sufficient	10	33.3	19	63.3	5.41	0.02*
**Has own income**						
No	20	66.7	25	83.3		
Yes	10	33.3	5	16.7	2.22	0.14
**Live**						
Alone	11	36.7	4	13.3		
With family	19	63.3	26	86.7	4.36	0.04*

(*) Statistically significant at *p* < 0.05.

As regards sleep quality, Table 2 indicates that all patients (100.0%) in both groups had poor sleep before the intervention, with no statistically significant difference in their total PSQI mean scores, 14.1 ± 3.8 and 13.2 ± 3.5 (*P* = 0.11). However, the EFT group had significantly higher scores in the PSQI dimensions of efficiency 2.5 ± 0.8 (*P* < 0.001), duration 2.0 ± 1.0 (*P* < 0.001), disturbance 2.1 ± 0.7 (*P* = 0.04), and latency 2.5 ± 0.8 (*P* < 0.001). Conversely, the sleep hygiene group had significantly higher scores in the PSQI dimensions of overall sleep 2.7 ± 0.5 (*P* < 0.001), medication 2.2 ± 1.1 use (*P* = 0.03), and daytime dysfunction 2.2 ± 0.7 (*P* < 0.001). After the intervention, 73.3% of the EFT group had good sleep quality, compared to 100.0% of those in the sleep hygiene group, and the difference was statistically significant (*P* = 0.005). Those EFT patients also had significantly higher scores in all PSQI dimensions except those of overall sleep (*P* = 0.01), and daytime dysfunction (*P* = 0.007), which were significantly higher in the sleep hygiene group.

**Table 2:** Sleep quality scores among elderly patients in the two study groups throughout the intervention.

PSQI scores (max = 3)	Group				Mann Whitney test	*p*-value
	EFT		Sleep hygiene			
	Mean ± SD	Median	Mean ± SD	Median		
**Per-intervention**						
Latency	2.5 ± 0.8	3.00	1.8 ± 0.4	2.00	16.39	< 0.001*
Duration	2.0 ± 1.0	2.00	0.7 ± 0.7	1.00	21.81	< 0.001*
Efficiency	2.5 ± 0.8	3.00	1.8 ± 0.4	2.00	16.39	< 0.001*
Disturbance	2.1 ± 0.7	2.00	1.8 ± 0.4	2.00	4.11	0.04*
Medications use	1.8 ± 0.9	2.00	2.2 ± 1.1	3.00	4.92	0.03*
	1.2 ± 0.4	1.00	2.2 ± 0.7	2.20	27.99	< 0.001*
Overall	2.0 ± 0.0	2.00	2.7 ± 0.5	3.00	29.50	< 0.001*
Total (max = 21)	14.1 ± 3.8	15.00	13.2 ± 3.5	14.50	2.52	0.11
Total PSQI						
Poor	30 (100.0%)		30 (100.0%)		–	–
**Post-intervention**						
Latency	1.4 ± 0.8	1.00	0.8 ± 0.4	1.00	12.92	< 0.001*
Duration	0.4 ± 0.8	0.00	0.0 ± 0.0	0.00	6.55	0.01*
Efficiency	1.1 ± 1.0	1.00	0.0 ± 0.0	0.00	28.38	< 0.001*
Disturbance	1.2 ± 0.5	1.00	0.8 ± 0.4	1.00	10.83	0.001*
Medications use	0.5 ± 1.1	0.00	0.2 ± 0.4	0.00	0.08	0.78
Daytime dysfunction	0.7 ± 1.1	0.00	0.8 ± 0.4	1.00	7.19	0.007*
Overall	0.7 ± 1.1	0.00	0.8 ± 0.4	1.00	6.04	0.01*
Total (max = 21)	5.9 ± 6.1	3.00	3.5 ± 1.6	4.00	0.47	0.49
Total PSQI						
Good	22 (73.3%)		30 (100.0%)			
Poor	8 (26.7%)		0 (0.0%)		Fisher	0.005*

(*)Statistically significant at *p* < 0.05.

Table 3 demonstrates that, at the pre-intervention phase, all patients in both groups had depression and low satisfaction with their life. The median scores of depression and life satisfaction were slightly lower in the sleep hygiene group 13.00 and 8.00 versus 14.00 and 11.00 in the EFT group, but the differences were not statistically significant. At the post-intervention phase, 33.3% of those in the EFT had depression, compared with 16.7% of those in the sleep hygiene group. Although this difference was not statistically significant, the median score of depression (3.00) was significantly (*P* < 0.001) higher in the EFT group in comparison with the sleep hygiene group (0.00). Similarly, 83.3% of those in the EFT had high life satisfaction, compared with 100.0% of those in the sleep hygiene group. This difference was not statistically significant. Meanwhile, in quantitative comparison, the median score of life satisfaction (30.00) was significantly (*P* = 0.007) higher in the EFT group in comparison with the sleep hygiene group (22.00).

**Table 3:** Life satisfaction, and sleep quality among elderly patients in the two study groups throughout the intervention.

	Group				X^2^ test	*p*-value
	EFT		Sleep hygiene			
	No	%	No	%		
**Pre-intervention**						
Have depression						
Yes	30	100.0	30	100.0	0.00	1.00
Depression score						
Range	7.0–14.0		8.0–14.0			
Mean ± SD	11.8 ± 3.0		12.3 ± 1.3		2.50	0.11
Median	14.00		13.00			
Satisfaction with life						
Low	30	100.0	30	100.0	0.00	1.00
Satisfaction score						
Range	5.0–14.0		5.0–14.0			
Mean ± SD	11.1 ± 3.1		10.4 ± 4.1		0.63	0.43
Median	11.00		8.00			
**Post-intervention**						
Have depression						
No	20	66.7	25	83.3		
Yes	10	33.3	5	16.7	2.22	0.14
Depression score						
Range	2.0–14.0		0.0–5.0			
Mean ± SD	4.9 ± 4.1		0.8 ± 1.9		29.20	< 0.001*
Median	3.00		0.00			
Satisfaction with life						
Low	5	16.7	0	0.0		
High	25	83.3	30	100.0	Fisher	0.052
Satisfaction score						
Range	10.0–30.0		22.0–31.0			
Mean ± SD	25.8 ± 7.4		23.7 ± 3.3		7.21	0.007*
Median	30.00		22.00			

(*)Statistically significant at *p* < 0.05.

The comparisons of the pre-post-intervention changes in each group (Table 4) illustrates statistically significant improvements in the levels of depression and life satisfaction, as well in their respective score (*P* < 0.001). Thus, the percentages of patients having depression dropped from 100% to 33.3% in the EFT group, and 16.7% in the sleep hygiene group. Similarly, those having life satisfaction with their life increased from 0.0% to 83.3% and 100.0% respectively. Moreover, the quality of sleep and the PSQI scores showed statistically significant improvements in both groups (*P* < 0.001). For instance, the mean scores of PSQI decreased from 14.1 to 5.9 in the EFT group and from 13.2 to 3.5 in the sleep hygiene group.

**Table 4:** Comparison of pre-post-intervention depression, life satisfaction, and sleep quality among elderly patients in the EFT and sleep hygiene education groups.

	Pre		Post		X^2^ test	*p*-value
	No	%	No	%		
**EFT group**						
**Have depression**						
No	0	0.0	20	66.7		
Yes	30	100.0	10	33.3	30.00	< 0.001*
**Depression score**						
Range	7.0–14.0		2.0–14.0			
Mean ± SD	11.8 ± 3.0		4.9 ± 4.1		U = 28.64	< 0.001*
Median	14.00		3.00			
**Satisfaction with life**						
Low	30	100.0	5	16.7		
High	0	0.0	25	83.3	42.86	< 0.001*
**Satisfaction score**						
Range	5.0–14.0		10.0–30.0			
Mean ± SD	11.1 ± 3.1		25.8 ± 7.4		U = 24.45	< 0.001*
Median	11.00		30.00			
**Sleep PSQI**						
Good	0	0.0	22	73.3		
Poor	30	100.0	8	26.7	34.74	< 0.001*
Range	6.0–17.0		2.0–19.0			
Mean ± SD	14.1 ± 3.8		5.9 ± 6.1		U = 18.65	< 0.001*
Median	15.00		3.00			
**Sleep hygiene education group**						
**Have depression**						
No	0	0.0	25	83.3		
Yes	30	100.0	5	16.7	42.86	< 0.001*
**Depression score**						
Range	8.0–14.0		0.0–5.0			
Mean ± SD	12.3 ± 1.3		0.8 ± 1.9		U = 48.98	< 0.001*
Median	13.00		0.00			
**Satisfaction with life**						
Low	30	100.0	0	0.0		
High	0	0.0	30	100.0	60.00	< 0.001*
**Satisfaction score**						
Range	5.0–16.0		22.0–31.0			
Mean ± SD	10.4 ± 4.1		23.7 ± 3.3		U = 46.29	< 0.001*
Median	8.00		22.0			
**Sleep PSQI**						
Good	0	0.0	30	100.0		
Poor	30	100.0	0	0.0	60.00	< 0.001*
Range	6.0–16.0		0.0–5.0			
Mean ± SD	13.2 ± 3.5		3.5 ± 1.6		U = 46.51	< 0.001*
Median	14.50		4.00			

(*)Statistically significant at *p* < 0.05, (U) Mann Whitney test.

Table 5 points to statistically significant strong negative correlations between patients’ scores of life satisfaction and each of the depression (r = −0.731) and PSQI (r = −0.808) scores. Additionally, a statistically significant strong positive correlation was revealed between patients’ scores of depression and PSQI (r = 0.766). The table also shows statistically significant weak negative correlations between patients’ scores of life satisfaction and their educational level (r = −0.227) and income (r = −0.317).

**Table 5:** Correlation matrix of elderly patients overall scores of depression, life satisfaction, and PSQI and their characteristics.

	Spearman's rank correlation coefficient		
	Depression	Satisfaction	PSQI
Depression	1.000	−.731**	.766**
Satisfaction	−.731**	1.000	−.808**
PSQI	.766**	−.808**	1.000
Age	−.007	−.002	.038
Educational level	.071	−.227*	.137
Income	.134	−.317**	.150

(*)Statistically significant at *p* < 0.05, (**)statistically significant at *p* < 0.01.

The multivariate analyses (Table 6) identified the study intervention as the main statistically significant negative predictor of the PSQI score, in addition to being in the sleep hygiene group, and having sufficient income. Conversely, having a university-level education was a positive predictor. As indicated by its standardized beta coefficient (−0.74), the intervention was the most influencing factor on this score, while the type of intervention had less but significant influence (−0.20). The model explains 63% of the change in the PSQI score.

**Table 6:** Best fitting multiple linear regression model for the PSQI, depression, and life satisfaction scores.

	Unstandardized coefficients		Standardized coefficients	t-test	*p*-value	95% confidence interval for B	
	B	SE				Lower	Upper
**PSQI score**^a^							
Constant	25.54	1.50		17.034	< 0.001	22.57	28.51
Intervention	−8.95	0.69	−0.74	13.050	< 0.001	−10.31	−7.59
Sleep hygiene group	−2.43	0.72	−0.20	3.354	0.001	−3.86	−0.99
University degree	5.31	1.23	0.43	4.333	0.000	2.88	7.74
Income	−2.72	1.19	−0.22	2.287	0.024	−5.08	−0.36
**Depression score**^b^							
Constant	20.54	1.38		14.929	< 0.001	17.81	23.27
Intervention	−5.80	0.51	−0.52	11.309	< 0.001	−6.82	−4.78
Sleep hygiene group	−2.19	0.39	−0.20	5.653	< 0.001	−2.96	−1.42
Married	1.57	0.42	0.14	3.689	< 0.001	0.72	2.41
Rural residence	−1.71	0.53	−0.15	3.248	0.002	−2.76	−0.67
Working	3.72	1.38	0.09	2.699	0.008	0.99	6.45
Income	3.79	0.45	0.34	8.495	< 0.001	2.91	4.67
Living with family	−2.54	0.58	−0.20	4.392	< 0.001	−3.68	−1.39
PSQI score	0.38	0.04	0.41	8.662	< 0.001	0.29	0.46
**Life satisfaction score**^c^							
Constant	11.16	2.18		5.111	< 0.001	6.83	15.49
Intervention	9.29	1.00	0.55	9.290	< 0.001	7.31	11.27
University education	3.41	1.23	0.19	2.783	0.006	0.98	5.84
Working	−8.19	2.58	−0.12	3.177	0.002	−13.29	−3.08
Income	−7.55	1.16	−0.44	6.521	< 0.001	−9.85	−5.26
PSQI score	−0.53	0.08	−0.38	6.237	< 0.001	−0.70	−0.36

^a^R-square = 0.63, Model ANOVA: F = 49.64, *p* < 0.001.

Variables entered and excluded: age, gender, marital status, residence, job status, living alone.

^b^R-square = 0.89, Model ANOVA: F = 123.55, *p* < 0.001.

Variables entered and excluded: age, gender, education, residence.

^c^R-square = 0.83, Model ANOVA: F = 114.04, *p* < 0.001.

Variables entered and excluded: age, gender, marital status, residence, living alone, depression score, group.

As regards the depression score, the table shows that the study intervention was its main statistically significant negative predictor, in addition to being in the sleep hygiene group, residing in rural areas, and living with the family. Conversely, being married, working, having sufficient income, as well as the PSQI score, were positive predictors. As shown by its standardized beta coefficient (−0.52), the intervention was the most influencing factor on the depression score, while the type of intervention had less but significant influence (−0.20). The model explains 89% of the change in the depression score.

The table also demonstrates that the study intervention and having a university-level education were statistically significant positive predictors of the life satisfaction score. On the other hand, working, having sufficient income, as well as the PSQI score, were negative predictors. As its standardized beta coefficient (0.55), the intervention was the most influencing factor on the life satisfaction score, whereas the type of intervention had no significant influence. The model explains 83% of the change in this score.

## Discussion

The study results indicate that both SHE and EFT approaches are effective in improving elderly patients’ sleep quality, as well as their depression symptoms and life satisfaction. The effects on sleep quality and depression are significantly higher among those in the SHE, leading to acceptance of the first and second research hypotheses.

Before implementation of the present study intervention, all the elderly in the two groups had poor quality sleep and suffered from depression symptoms. A high level of depression is quite expected given its close association with the lack of good sleep. Thus, a strong positive correlation was revealed between the scores of poor quality sleep (PSQI) and depression. Moreover, the PSQI score was identified as a significant independent positive predictor of the depression score. In agreement with this, a study in China in their study of sleep and depression in the elderly showed that sleep quality is a significant risk factor of depression^30^. Similarly, a study in Nepal revealed that depression was an underlying cause of unrecognized depression among elderly people^31^.

Moreover, the majority of the present study elderly patients had low satisfaction with life at the pre-intervention phase. This could partly be attributed to their poor sleep and high depression. This is supported by the strong negative correlations between the life satisfaction score and each of the PSQI and depression scores. In addition, the PSQI score turned out to be a significant independent negative predictor of the life satisfaction score. In line with this, a study in Sweden demonstrated decreases in life satisfaction scores with increasing age^32^.

The implementation of the current study intervention led to significant improvements in the sleep quality in both study groups, as well as in their depression symptoms and life satisfaction. Moreover, the intervention was identified as a significant independent negative predictor of the PSQI and depression sores, and a positive predictor of the life satisfaction score. These findings indicate that both approaches are effective in the management of sleep problems among these elderly patients, with positive impacts on their depression symptoms as well as on their satisfaction with life. Similar positive effects of sleep hygiene education on the quality of sleep were reported in recent reviews^33,34^.

Although the current study intervention was associated with significant improvements in the sleep quality in both study groups, the effect was more prominent in the SHE group. Thus, all elderly patients in the SHE group regained a good quality sleep compared with approximately three-fourths of the EFT group, indicating superiority of this former approach and confirming the first set hypothesis. Moreover, being in the SHE group was a significant independent negative predictor of PSQI score, indicating better sleep quality. This difference could be attributed to the nature of both approaches as; the SHE approach is more familiar for the elderly in comparison with the tapping maneuver in the EFT approach. In congruence with this, a study in the United States showed more participants’ preference of SHE strategies to treat insomnia^35^. On the other hand, the literature shows more use of SHE in sleep problems while EFT is more used for the relief of anxiety and depression^36,37^.

The second research hypothesis in the current study postulated that the elderly patients receiving the SHE intervention will have less depression in comparison with those receiving EFT intervention. The results also lead to acceptance of this hypothesis as the post-intervention depression score was significantly higher in the EFT group compared with the SHE group. Additionally, the multivariate analysis demonstrated that being in the SBE group was a significant independent negative predictor of the depression score. This might be explained by the more improvement in their sleep quality and its lessening effect on their depression symptoms. In agreement with this, Silva et al.^38^ in a study in Brazil concluded that depressive symptoms could be improved with the *management* of sleep problems. Meanwhile, a study in China demonstrated that an intervention to improve elderly patients’ depression led to improvement in their sleep quality^39^. Hence, a reciprocal relation could be claimed between sleep quality and depression.

Concerning life satisfaction, the post-intervention results of the current study indicate no statistically significant difference in the categorical comparison, but the quantitative comparison demonstrated that the score was significantly higher in the EFT group compared with the SHE group. However, the multivariate analysis could not identify an independent group effect on the life satisfaction score, but the intervention was a positive predictor of this score. Therefore, the third research hypothesis could be refuted. Nonetheless, a study in Canada demonstrated a close association between sleep hygiene and life satisfaction^40^.

Other personal characteristics had significant and independent influences on the post-intervention scores of the three present study outcomes. Thus, the elderly patients with university level of education had less benefit from the intervention on their sleep quality, but more benefit related to life satisfaction. This might be explained by the finding that life satisfaction is negatively correlated to the life satisfaction score. Thus, those with a university-level of education tended to have lower life satisfaction scores at the baseline, and thus showed more improvement following the intervention. The effect of educational attainment on sleep has been shown in a study in Indonesia^35^ and on life satisfaction in a study in the United States^41^.

Other socio-demographic characteristics had variable effects on elderly patients’ sleep quality, depression, and life satisfaction. These include marital status, living with family, working, income, and place of residence. Some of these characteristics were significantly different between the two groups such as education, income, and living with family. As they could confound the effect of the intervention, multivariate analyses were carried out to adjust for any such effects.

The main study limitation was the design being open-label, with both patients and researchers being aware of the group allocation. This could not be concealed given the nature of the intervention. However, the assessment of the study outcomes was blind to avoid the bias of open-label design.

## Conclusion and recommendations

The findings point to the beneficial effects of each of the SHE and EFT approaches on elderly patients’ sleep quality. The SHE approach is more effective in ameliorating the quality of sleep.

The study recommends periodic screening of sleep quality using the PSQI scale on elderly patients in various healthcare settings as well as in nursing homes and implementation of the SHE approach to those with diagnosed disorders. Geriatric nurses need to be trained in administering this program. Future replication of this study is needed on a large probability sample from different geographical areas to help for the generalization of the results.

### Ethical statement

The current study was approved by the Ethical Committee at the Faculty of Medicine, Alexandria University, Egypt; approval number (0,305,317). The clinical trial was registered in the “Clinical Trials.gov Protocol Registration and Results System (PRS)”; clinical trial registration number is (0,305,317/2021) and the full date of first registration is 19/ 02 / 2022. Available at: https://beta.clinicaltrials.gov/study/NCT05276635?patient=Effectiveness%20of%20Emotional%20Freedom%20Techniques%20vs%20Sleep%20Hygiene%20Education%20Group%20Therapy%20(SHE)%20in%20Management%20of%20Sleep%20Disorders%20Among%20Elderly&locStr=&distance=0

### Guidelines

All methods were carried out by relevant guidelines and regulations.

## Data Availability

The datasets used and/or analyzed during the current study are available from the corresponding author on reasonable request.
